# Dr. Gordon on Clinical Medicine

**Published:** 1829-07-01

**Authors:** 


### Clinical Medicine.

We are glad to see that this neglected branch of medical instruction, though the most important of all, is gradually lifting its head, and forcing itself on the attention of teachers and "public functionaries." Dr. Gordon, of the London Hospital, a most zealous and talented physician, has published an Introductory Lecture to his Course of Clinical Instruction, which we recommend to the perusal of all medical officers of public institutions. It contains sound principles and most judicious advice—not only for the teacher but the pupil. We can only offer an extract, as a sample of the lecture. After exposing the fatal errors of wild hypothesis, the able author brings in the empiric and the routinist for their share of censure. In truth, we have good reason to know, that practice without principles does infinitely more harm, because it has a much greater sphere for its action, than principles without practice!

"Let us now reverse the picture; and if I have spoken with some severity of mere hypothesis, how much more keenly might we depict 'practice without principles?' How should we catch the ever varying lineaments of the many-headed monster 'empiricism,' in all its shades and gradations, from the man, not of one idea, but of one drug, up to the respectable and regular practitioner, who has a particular remedy for each and every disease, without knowing what the disease really is, or in what organ of the body it is seated?—the empirical knowledge preached and acted upon by nurses and *Lady Bountifuls*, and by practitioners who deserve to be ranked with them, confounding in their frightful confidence one disease with another, treating colic for enteritis, and rheumatism of the intercostals for pneumonia!

"The correction of both these errors is the great object of a clinical course, in which the attention of the student is directed to disease as it really exists; and thus supposing him to be, as he ought, acquainted with the principles of his profession, theory and practice are made mutually to aid and correct each other. Here he will also soon begin to learn a painful truth:—the uncertainty of his art, even when entered upon with every possible advantage; and understand the necessity which the conscientious practitioner feels of carrying along with him the principles by which a clinical course is regulated—the daily necessity of correcting his previous impressions, by the facts which come before him—of altering his confidence in the distinctive characters of disease, and the action of remedies. The uncertainty of our art, indeed, has not escaped the sneer of the philosopher, nor the sting of the humorist; and has been made the frequent theme of wit and ridicule. The following apologue, says D'Alembert, made by a physician, a man of wit and philosophy, represents, very well, the state of medicine:—'Nature is fighting with the disease—a blind man armed with a club, that is, the physician, arrives to settle the difference,—he first tries to make peace; when he cannot accomplish this, he lifts his club and strikes at random. If he strikes the disease, he kills the disease; if he strikes nature, he kills nature.' This story, which the humorist applies to the art itself, may not unaptly fit those who exercise it on unsound principles; and few of us there are who have not, in our experience, seen specimens of practice in which the random blows of the blind man may be recognized.

"I have thus endeavoured to point out the indispensable connexion of theory and practice, and clinical study as the only vinculum, or tie, by which they are to be united. I have adverted to the uncertainty of medicine, as a motive to perpetual exertion, and depicted our profession as one of daily study as well as daily toil. I have done this advisedly, feeling, that in addressing an audience of medical students, it is my duty, as it is my pride, to impress upon you the extent and dignity of a profession to excel in which, requires a greater compass of knowledge than any other art—a greater variety of liberal accomplishments—and a quicker and readier application of these to the wants, necessities, and afflictions of mankind."

---

